# *She* more than *he*: gender bias supports the empathic nature of yawn contagion in *Homo sapiens*

**DOI:** 10.1098/rsos.150459

**Published:** 2016-02-03

**Authors:** Ivan Norscia, Elisa Demuru, Elisabetta Palagi

**Affiliations:** 1 Museo di Storia Naturale, Università di Pisa, Via Roma 79, Calci, Pisa 56011, Italy; 2 Unità di Primatologia Cognitiva, ISTC-CNR, via Aldrovandi 16b, 00197 Roma, Italy

**Keywords:** yawn contagion, humans, empathy, female bias, social bonding

## Abstract

Psychological, clinical and neurobiological findings endorse that empathic abilities are more developed in women than in men. Because there is growing evidence that yawn contagion is an empathy-based phenomenon, we expect that the female bias in the empathic abilities reflects on a gender skew in the responsiveness to others’ yawns. We verified this assumption by applying a linear model on a dataset gathered during a 5 year period of naturalistic observations on humans. Gender, age and social bond were included in the analysis as fixed factors. The social bond and the receiver’s gender remained in the best model. The rates of contagion were significantly lower between acquaintances than between friends and family members, and significantly higher in women than in men. These results not only confirm that yawn contagion is sensitive to social closeness, but also that the phenomenon is affected by the same gender bias affecting empathy. The sex skew, also found in other non-human species, fits with the female social roles which are likely to require higher empathic abilities (e.g. parental care, group cohesion maintenance, social mediation). The fact that female influence in social dynamics also relies on face-to-face emotional exchange raises concerns on the negative repercussions of having women’s facial expressions forcibly concealed.

## 1. Introduction

Empathy is defined as the ability to understand and share the internal states of others [1]. This ability is vital to engage in successful relationships within complex social networks and, consequently, to increase individual fitness [2]. Possibly, because women are hard-wired for maternity and parental care, they have been classically considered as more empathic than men (for an extensive review, see [3]). Psychological studies indeed report that women score higher than men on different self-reported measures of empathy in childhood [4], adolescence [5,6] and adulthood [7–9] with differences growing with age during the puberty period [5,10–13].

The higher empathic capacity of women is also strongly suggested by neurobiological studies focusing on the mirror neuron system. Through the recruitment of this system, an observer can preconsciously activate shared emotional representations during the perception of an action or of a facial expression of others [14–16]. This activation response, known as perception–action mechanism [14], is a basic requirement of empathy because it allows individuals to automatically experience others’ affective states [3,14,17]. Empathy is considered to be the result of the interactions between mirror neuron areas and emotional-processing brain centres [3,18].

Through a study of functional magnetic resonance imaging, Schulte-Rüther *et al*. [19] found that women activated more than men the inferior frontal cortex when asked to focus on either their own feelings or the feelings of another person while seeing facial emotional expressions. Such brain area was found to include mirror neurons, as it had been previously hypothesized [20,21]. Moreover, within the same brain area (inferior frontal gyrus, *pars opercularis*) women seem to possess larger grey matter volume compared with men, with the larger grey matter volume being also coupled with higher self-reported scores in the emotional empathic propensity [22].

One of the outputs of the perception–action coupling involving the mirror neuron system is facial mimicry which, in turn, is positively associated with empathy [23,24]. Measures of facial electromyography revealed greater facial muscle reactivity in women, compared with men, when exposed to facial expressions of anger and happiness [25,26]. Additionally, women rely more than men on facial feedback for recognizing facial expressions [27].

Hence, it is not surprising that one behavioural manifestation of empathy is facial mimicry, including contagious yawning [3,28,29].

Yawning is an involuntary sequence of mouth opening, deep inspiration, brief apnoea and slow expiration. In humans, yawns last on average 6 s, and the individual yawn duration and frequency remains remarkably stable over weeks [30]. Yawning is an interesting topic for neurobehavioural research owing to its implications in several neuroendocrine and physiological activities including sleep–awake rhythms, thermoregulation, vigilance and consequently, in the diagnostic of related disorders (for an extensive review, see Walusinski [31]). Possibly because of its strict association with hormones and physiology, yawning performance can vary as a function of the degree of sexual dimorphism (*sexual dimorphism hypothesis*, [32]). For example, in rhesus macaques, yawning rates are strictly linked to testosterone levels, thus being more frequent in males than in females [33], the same occurs in geladas [34]. In humans, Schino & Aureli [35] noted that such androgen-driven dimorphism is not present, with men and women yawning equally often.

Yawning is contagious in that it can be triggered by others’ yawns [30]. A wide range of sensory modes are vectors of contagious yawning in humans, ranging from hearing [36], seeing [30,37], reading about [30] or even thinking about yawning [30]. Moreover, yawn contagion in humans can be affected by different variables, such as the time of the day [38], age [39] or familiarity between subjects [40]. As a physiological response, yawn contagion is expected to be sensitive to the interaction between individual, environmental and social factors. Despite few controversial results [39], there is growing evidence that yawn contagion is an empathy-based phenomenon. Contagious yawning recruits different neuronal networks involved in empathic processing, including the inferior frontal gyrus and other mirror neuron areas [36,41–45]. Although some aspects of empathy may appear earlier than others [46], contagious yawning follows a similar ontogenetic trajectory as empathy. It increases with age starting at 4–5 years [47,48] when the ability to identify others’ emotions is being acquired [2,49,50] and declines with old age [39] when empathic abilities also decline [51]. Contagion is significantly less likely in subjects suffering from empathy disorders, such as autism and psychopathy [52–55]. Contagious yawning follows an empathy gradient (*sensu* [14]) being more frequent in response to kin, then friends, then acquaintances, and lastly strangers [40].

Previous reports indicate that not all individuals are susceptible to others’ yawns. Approximately 40–60% of healthy humans were never observed yawning in response to a yawn stimulus under laboratory conditions [28,30,37]. Moreover, susceptibility to others’ yawns appears to be stable under different experimental contexts, and yawn susceptibility is not significantly different between men and women [39]. In their naturalistic study on yawn contagion, Norscia & Palagi [40] considered all the potential responders, which also included subjects showing no contagion. The authors found that the probability to contagiously yawn was affected by social bond more than by any other tested variable, including gender. Therefore, social modulation more than individual features appeared to affect the probability to respond to others’ yawns under natural settings. Yet, within the susceptible population, the level of yawn contagion may also vary according to different individual features. If yawn contagion is an empathy-based phenomenon, then we expect social bond to be confirmed as a variable that significantly affects yawn contagion frequencies (prediction 1a). Moreover, if women are more empathic than men, then we also expect that in the susceptible population women are infected at higher rates by others’ yawns compared with men (prediction 1b). We verified these assumptions through an ethological, naturalistic approach based on a 5 year period (2010–2015) of direct observation on humans.

## 2. Methods

### 2.1 Data collection

Data were gathered from the 26 October 2010 to the 27 April 2015, from 07.00 to 02.00, on human males and females in their natural environment, e.g. in office, over dinner, during social events, etc., with the individuals being unaware of being observed and in absence of any evident external source of anxiety. This 5 year dataset includes the 1 year dataset used by Norscia & Palagi [40]. The study subjects (greater than or equal to 17 years of age) and their personal data (e.g. age, lack of reported empathy-related pathologies, relationship with others) were known by the authors. Depending on the circumstances, the information was recorded, unnoted, through alphanumerical codes and entered directly into calculation sheets, typed in mobile phones or written on notebooks and then copied in calculation sheets for data analysis.

Previous literature reports that yawn responses can be elicited within 5 min after watching someone else’s yawn (the trigger’s yawn) [30], with a maximum in the first minute [56,57]. Following Norscia & Palagi [40], we recorded yawn responses within a 3 min time window, thus reducing the probability of mistakenly coding spontaneous yawns as yawn responses. Moreover, we excluded minute four and five from the analyses to reduce the probability of mistakenly coding a yawn response as a spontaneous yawn. Hence, we coded a yawn as ‘spontaneous’ when no other subject had yawned in the 5 min preceding the yawning event. All the potential yawners (men and women) had to be within the range of the stereoscopic view of each observer. The three authors collected the data and before starting systematic data collection, interobserver reliability was tested during a 10 day concurrent trial period, until Cohen’s kappas (*k*) were greater than or equal to 0.75 [58].

### 2.2 Dataset and statistical analysis

Statistical tests were carried out via SPSS 20.0. Data distributions were tested for normality via EasyFit 5.6. At the end of the observation period (closed for these analyses on the 27 April 2015), our dataset included 1461 yawning bouts. To verify whether human females spontaneously yawned significantly more than males, we restricted the analysis to 104 spontaneous yawners (triggers) observed for at least 120 min, so to avoid any observational bias and ensure the sufficient observation time necessary to ascertain the yawning propensity (in terms of frequency) of each subject. Owing to non-normality of data distribution (Anderson–Darling test: *n*_men_=48;*p*<0.01; *n*_women_=56,*p*<0.01), the comparison of spontaneous yawn frequency between sexes was tested via Mann–Whitney’s non-parametric test for independent samples.

Regarding yawn contagion, we excluded pairs of strangers (yawn trigger and observer) from the dataset, because by definition, they were subjects meeting for the first time. This condition did not allow subjects’ follow-up and the possibility to calculate yawning frequencies across time. Moreover, we eliminated the bouts related to multiple triggering yawns emitted by different subjects, to ensure that the yawn response was undoubtedly elicited by a specific trigger. After this first data selection, we further restricted the analysis to 92 dyads, meeting the following criteria: (i) yawn contagion was present, so the dyad was composed by the yawn trigger and a responder (susceptible subject), and (ii) at least three independent occasions of contagion had to be available, to make sure that yawn contagion susceptibility was correctly detected and that possible low response frequencies were not owing to insufficient data availability. Independent occasions required the trigger spontaneous yawns to be spaced more than 5 min apart from one another.

For each pair of yawn trigger and responder, the yawn contagion frequencies were calculated as the number of yawn responses weighed on the number of independent occasions. According to Norscia & Palagi [40], three levels of social bond were recognized: 1= acquaintances, who frequented each other because of a third common element, namely school (classmates), work (colleagues) or a friend in common (friends of friends); 2= friends, unrelated subjects frequenting each other on a totally voluntary basis, in the absence of any third element forcing them to meet; 3= regular partners and strictly related individuals (kin; *r*>0.25). Dubious cases were not included in the dataset (e.g. friends who had had a previous sentimental relationship, etc.).

After verifying the normal distribution of the target variable (Anderson–Darling test: *n*_dyads_=92;*p*= n.s.), we ran a linear model to examine the effect of different fixed factors on the frequency of yawn contagion (target, scale variable) of the individuals who responded to others’ yawns at least once. The fixed factors included in the model are: social bond (nominal variable; from 1 to 3 depending on the social closeness), trigger’s and responder’s sex (nominal variables: male =1, female =0), and trigger’s and responder’s age (scale variables).

We tested the model for each combination involving the variables of interest, spanning from the null model (intercept only) to a model including all the fixed factors (full model). The model with the lowest value of Akaike’s corrected information criterion (AICc) was considered to be the best model. Via the multivariate regression model, we calculated the effect size of each variable remaining in the best model.

## 3. Results

No significant difference in spontaneous yawn frequencies was found between men and women (Mann–Whitney’s test: *n*_men_=48; *n*_women_=56; *U*=1305.50;*p*=0.785). Moreover, 34.5% of the people exposed to at least three others’ spontaneous yawns were actual responders because they returned at least one yawn within the 3 min time slot.

**Figure 1.**
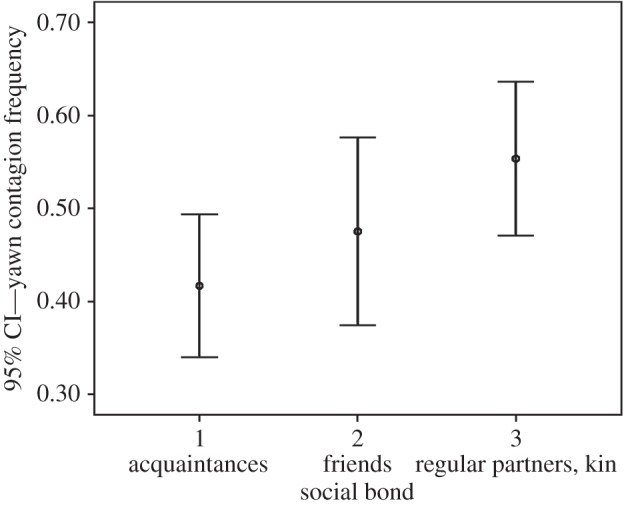
Yawn contagion frequency (mean and 95% confidence interval (CI)) as a function of the social bond shared by the subjects.

**Figure 2.**
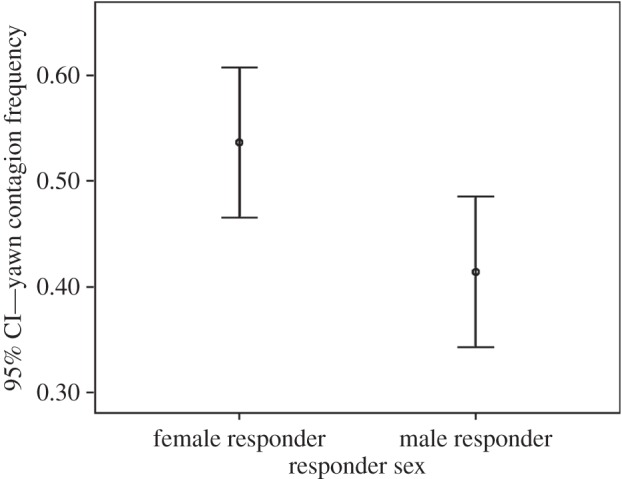
Yawn contagion frequency (mean and 95% CI) as a function of the sex of the responder.

Via the linear model, we evaluated whether social bond, yawn trigger’s sex and age, and receiver’s sex and age significantly influenced yawn contagion frequency within actual responders. Both the social bond and the receiver’s sex (but not their interaction) remained in the best model (best model AICc: 7.781; worst model AICc: 31.248) and had a significant effect on yawn contagion rates (table 1). The effect size is intermediate to large (sex responder: Cohen’s *d* corrected for the different sample sizes =0.513; bond: Cohen’s *d* for multiple groups: 0.788). In particular, the rate of contagion was lowest between acquaintances than between friends and family members (figure 1). Moreover, women responded more frequently than men to yawning stimuli (figure 2).

**Table 1.** Best linear model (AICc =7.781) explaining yawn contagion frequency as a function of the social bond between yawn trigger and responder (*F*=3.938, d.f. 1=2, d.f. 2=88, *p*=0.023) and receiver’s sex (*F*=7.371, d.f. 1=1, d.f. 2=88,*p*=0.008). Intercept result: *F*=4.751, d.f. 1=3, d.f. 2=88,*p*=0.004.

factors	coefficient	s.e.	*t*	significance level	confidence interval 95%
intercept	0.491	0.045	10.917	0.000	0.402/0.581
social bond (1)	−0.147	0.052	−2.802	0.006	−0.250/−0.043
social bond (2)	−0.093	0.078	−1.200	0.233	−0.247/0.061
social bond (3)	0.000^a^				
responder’s sex (f)	0.132	0.049	2.715	0.008	0.035–0.229
responder’s sex (m)	0.000^a^				

^a^Redundant coefficients.

## 4. Discussion

Our results show that in the individuals that are susceptible to yawn contagion, the rates of yawn responses are affected by both the social bond linking trigger and responder and by responder’s gender. In particular, yawn contagion rates were significantly lower between acquaintances than between friends and family members (prediction 1a confirmed; figure 1) and women responded at higher rates than men (prediction 1b confirmed; figure 2) even though men and women have not been found to differ in their rates of spontaneous yawning ([35]; this study).

The former result is consistent with previous findings by Norscia & Palagi [40], who reported that in natural conditions the occurrence and frequency of yawn contagion correlated with the level of social closeness. The relationship quality (acquaintances, friends and kin) significantly explains the variation of yawn contagion in humans, either considering all the potential responders [40] or susceptible subjects only (present study). The increase of yawn contagion rates along with social attachment supports the hypothesis that this phenomenon has an empathic basis. In fact, one outcome of the perception–action model [14] is that the more compatible and socially tied two subjects are, the easier interpartner identification is [59]. Yawn contagion is socially modulated also in non-human primates. Chimpanzees (*Pan troglodytes*) contagiously yawn more in response to in-group compared with out-group members [60]. In bonobos (*Pan paniscus*), yawn contagion peaks among closely bonded individuals, which are those who exchange more affinitive contacts [61]. A comparative study analysed the variation in yawn contagion in humans and bonobos and showed that its rates were affected by the social bond more than by the species, thus highlighting the salience of interindividual attachment to yawn contagion [57].

The relationship between yawn contagion and social bond is not limited to humans, bonobos and chimpanzees that share a close common ancestor (about 5–7 Myr ago) [62]. In geladas (*Theropithecus gelada*), yawning is especially contagious between socially close individuals [63], thus suggesting that yawn infectiousness may be an empathy-based phenomenon also in monkeys. This is in line with the bottom-up perspective proposed by de Waal & Ferrari [64], who posit that a cognitive continuity bridges non-human to human primates.

Although at variable frequencies, yawn contagion has been also described between dogs, wolves and between dogs and humans [65–70], O’Hara & Reeve [71] found no association between yawn response and the familiarity of the human models. Later Silva *et al*. [67] and Romero *et al*. [68] found that adult dogs yawned more in response to familiar than unfamiliar yawners, regardless of the sensory modality through which the animals perceived the stimulus (hearing or seeing). Therefore, there is evidence that yawn contagion between humans and dogs underlies some empathic abilities. In wolves, yawn contagion between conspecifics was also associated with the social closeness of group members [70]. Hence, in canids, familiarity or social bond can positively affect the frequency of yawning responses, suggesting that the susceptibility of yawn contagion might correlate with the level of emotional proximity [68,70].

The available data on social primates and canids are silent on whether the association between yawn contagion and emotional closeness found in these two mammalian taxa may have a common origin (homology) or be the outcome of convergent evolution related to social living (analogy). Whatever the case, empathy may be adaptive in highly cooperative and cognitively demanding social systems. In fact, empathy favours prosocial behaviour and dyadic closeness [3,72]. Through transitive emotional transmission [73], interindividual attachment can spread within the social network and increase group cohesion and cooperation.

The completely new finding of this study is that under natural conditions the women from our population sample contagiously yawned at significantly higher rates than men (figure 2). This result further supports the empathic ground of yawn contagion, in the light of the existing psychological, clinical and neurobiological evidence in favour of higher empathic abilities of women compared with men [4–9,19,22,25,26,74]. A recent study on humans found no relationship between empathic abilities or gender and yawn contagion. Yet, this study was conducted in laboratory conditions on a population including an enriched cohort of university students (mean age =32.0±15.7 s.d., range = 18–83 years) and was based on yawn video stimuli, self-reported contagion and self-reported scores for empathy [39]. It is not possible to make direct comparisons with our study, which is based on a different target population (with no prevalence of a specific cohort; mean age =41.7±11.3 s.d., range = 16–72 years), direct observations of people in their natural settings, not aware of being under study and responding to real stimuli. Moreover, we used the social linkage as a proxy for empathy at dyadic level, because although the empathic sensitivity can vary from one subject to another, the individual expression of empathy is strongly affected by the emotional bond shared by the subjects [14].

The literature examining sex differences in empathy-based behaviours is scarce but still suggests that—compared with males—females are more sensitive to others’ emotions and more inclined to behave prosocially [3]. For example, compared with males, female rats showed greater sensitivity to other’s pain (measured via an increase of writhing; [75]) and were more likely to release a trapped cagemate [76]. In chimpanzees, female bystanders were more likely to console-distressed individuals [77] and in lowland gorillas, immature females offered more frequently consolatory contact than males [78].

The presence of a female skew in the phenomenon of yawn contagion was detected in different non-human mammals and can be interpreted in the light of the role of females according to species-specific social dynamics. Romero *et al*. [70] found that female wolves showed a shorter reaction time than males when observing yawns of close associates, suggesting that females are more responsive to emotional, social stimuli. This may be possibly related to the fact that wolf family packs possess a division-of-labour system in which the female predominates primarily in such activities as defence and pup care [79] requiring the ability to quickly detect the emotional state of the offspring (e.g. distress, danger) and react accordingly.

In bonobos, Demuru & Palagi [61] found that group members would respond more likely to a female than to a male model. Also in this case, the role of females is crucial to interpret the result. In bonobos, adult females represent the relational and decisional nucleus of the society [80–84], thus playing a key role in affecting the emotional states of others [61,85].

In geladas, Palagi *et al*. [63] found a stronger and more specific matching of yawn types in female–female compared with female–male dyads. In this species, females form coalitions and long-term relationships, support each other in infant rearing and remain together, regardless of whether a dominant male is present or not [86–88]. According to these authors, the role of gelada females in cementing the group may rely on their capacity of being emotionally tuned to one another. The empathy gender bias suggested by yawn contagion provides biological and ethological support to some sociology studies that are revisiting the role of women in the mediation of social conflicts. For example, women as peace negotiators seem to be more generous and egalitarian than men in that they expect and ask for less. The propensity to fairness makes women potentially more successful to resolve disputes when equity is crucial to reach stable agreements, as it occurs in international conflicts involving disadvantaged parties [89,90].

Empathy enhances parental care, interindividual communication and group living, by motivating prosocial behaviours and favouring the development of moral reasoning [72]. The higher empathic abilities of women compared with men, also revealed by the gender bias in yawn contagion, may have social repercussions. The ability to preconsciously decode and replicate the emotions of others, e.g. via yawn contagion and facial mimicry, may allow women to respond with more appropriate behaviours toward others and to be more successful in forming enduring alliances [3,91]. What happens when women’s social influence is reduced by forcibly preventing them from decoding facial expressions or auditory signals to connect with others?

## Supplementary Material

supporting data.docx

## Supplementary Material

data_spontaneous_yawn_MF.xlsx

## Supplementary Material

database_GLMM.sav

## Data accessibility

The data used for this study are provided as electronic supplementary material to this article. The statistical tools EasyFit and SPSS used in this study are proprietary software. However, trials (limited time/limited functionality) can be found online on the producer’s websites (EasyFit: www.mathwave.com; SPSS: https://www-01.ibm.com).
