# Supplementary material for: She more than he: gender bias supports the empathic nature of yawn contagion in Homo sapiens
Source: R Soc Open Sci. 2016 Feb 3;3(2):150459. doi: 10.1098/rsos.150459 (PMC4785969; doi:10.1098/rsos.150459)
Supplement: supporting data.docx [file rsos150459supp1.docx]

Data accessibility. The datasets supporting this article have been uploaded as part of the electronic supplementary material.

Funding statement. This research has been supported by personal funding.

Authors’ contributions. I.N., E.D., E.P. conceived the study; I.N., E.D., E.P. collected data; I.N. and E.P. performed the analysis and wrote the first draft.

Conflict of interests. We have no competing interests.
